# *Helicobacter pylori* Promotes Epithelial–Mesenchymal Transition in Gastric Cancer by Downregulating Programmed Cell Death Protein 4 (PDCD4)

**DOI:** 10.1371/journal.pone.0105306

**Published:** 2014-08-21

**Authors:** Han Yu, Jiping Zeng, Xiuming Liang, Wenfu Wang, Yabin Zhou, Yundong Sun, Shili Liu, Wenjuan Li, Chunyan Chen, Jihui Jia

**Affiliations:** 1 Department of Microbiology/Key Laboratory for Experimental Teratology of Chinese Ministry of Education, School of Medicine, Shandong University, Jinan, PR China; 2 Departments of Biochemistry, School of Medicine, Shandong University, Jinan, PR China; University of Alabama at Birmingham, United States of America

## Abstract

*Helicobacter pylori*, a Gram-negative, microaerophilic bacterium found in the stomach, is assumed to be associated with carcinogenesis, invasion and metastasis in digestive diseases. Cytotoxin-associated gene A (CagA) is an oncogenic protein of *H. pylori* that is encoded by a Cag pathogenicity island related to the development of gastric cancer. The epithelial–mesenchymal transition (EMT) is the main biological event in invasion or metastasis of epithelial cells. *H. pylori* may promote EMT in human gastric cancer cell lines, but the specific mechanisms are still obscure. We explored the underlying molecular mechanism of EMT induced by *H. pylori* CagA in gastric cancer. In our article, we detected gastric cancer specimens and adjacent non-cancerous specimens by immunohistochemistry and found increased expression of the EMT-related regulatory protein TWIST1 and the mesenchymal marker vimentin in cancer tissues, while programmed cell death factor 4 (PDCD4) and the epithelial marker E-cadherin expression decreased in cancer specimens. These changes were associated with degree of tissue malignancy. In addition, PDCD4 and TWIST1 levels were related. In gastric cancer cells cocultured with CagA expression plasmid, CagA activated TWIST1 and vimentin expression, and inhibited E-cadherin expression by downregulating PDCD4. CagA also promoted mobility of gastric cancer cells by regulating PDCD4. Thus, *H. pylori* CagA induced EMT in gastric cancer cells, which reveals a new signaling pathway of EMT in gastric cancer cell lines.

## Introduction

*Helicobacter pylori* (*H. pylori*) is a helix-shaped, Gram-negative bacterium associated with digestive diseases, including chronic gastritis, peptic ulcer, gastric cancer, and mucosal-associated lymphoid tissue lymphoma. *H. pylori* has been classified as a carcinogen by the World Health Organization and the International Agency for Research on Cancer (WHO/IARC) in 1994 [1], [2].

*H. pylori* genes possess a cytotoxin-associated gene A (CagA) pathogenicity island that encodes the major virulence protein CagA and a type IV secretion system (T4SS). CagA is delivered to host cells by T4SS and induces cancer formation and progression [3]. *H. pylori* can deregulate cell proliferation, affect the normal apoptotic pathway, influence cell shape, abolish junctional activity and facilitate an epithelial-to-mesenchymal transition (EMT) phenotype after it translocates into the host cell [4], [5]. The *H. pylori* effector protein CagA affects most of these pathways. *In vivo* experiments showed that transgenic expression of CagA could cause multiple malignancies in mice, including gastric epithelial hyperplasia, myeloid leukemia and B-cell lymphomas, or gastric polyps and adenocarcinomas of the stomach and small intestine [6]. However, the molecular mechanism of *H. pylori* CagA interacting on host cells is still unclear.

The EMT was initially recognized as a feature of embryogenesis, a vital process for morphogenesis during embryonic and heart development. However, it also contributes to tissue fibrosis, tumor invasion and metastasis. EMT is characterized by several distinct traits, such as loss of cell adhesion, increased cell mobility, resistance to apoptosis, and some properties of stem cells. With EMT, epithelial markers such as E-cadherin show decreased expression and mesenchymal markers such as vimentin show increased expression. Other regulatory molecules such as SNAIL, TWIST and SLUG show changed expression [7], [8], [9]. Oncogenic pathways that may induce the EMT include transforming growth factor β (TGF-β), Src, Ets, Ras, Wnt/β-catenin, Notch, nuclear factor-κB and integrin [10], [11], [12].

Infection with the human pathogenic factor *H. pylori* is assumed to lead to EMT, invasion or metastasis of gastric cancer. For example, upregulation of matrix metalloproteinase 7 by pathogenic *H. pylori* partially depends on gastrin and may have a function in the development of gastric cancer, potentially through the EMT, by indirectly inducing levels of soluble heparin-binding endothelial growth factor [13]. *H. pylori* CagA is involved in invasion of tumor cells by deregulating c-met receptor signaling [14]. As well, CagA could increase the activation of the Wnt/β-catenin signaling pathway by disrupting the stabilization of the E-cadherin–β-catenin complex. Thus, CagA plays a vital role in the development of intestinal metaplasia [15]. In addition, microarray analysis suggested that the phosphorylation status of CagA may affect the expression of EMT-related genes in gastric cancer cells [16]. However, little is known about the mechanisms that underlie the EMT induced by CagA.

Programmed cell death protein 4 (PDCD4) is localized to the nucleus in proliferating cells [17]. As an important post-transcriptional target of microRNA (miR) miR-21, PDCD4 is related to tumor progression and prognosis in human lung, colorectal, breast and gastric cancer [18], [19]. The tumor suppressor PDCD4 can bind to eIF4A or eIF4G and inhibit translation. PDCD4 can regulate mitogen-activated protein 4 kinase 1 or urokinase plasminogen activator receptor (uPAR) expression to inhibit carcinoma invasion and intravasation [20], [21]. PDCD4 could also influence the transcription of genes. It interacts with the DNA binding domain of TWIST1 and inhibits Y-box binding protein 1 expression [22]. As well, loss of PDCD4 expression is associated with malignant transformation in gastric cancer [23], [24]. Downregulation of PDCD4 is related to the tumor differentiation in digestive tract cancers [25]. However, whether PDCD4 is involved in the EMT induced by CagA in gastric cancer and the specific mechanism still need further elucidation.

Here, we explored the regulation of EMT-associated proteins and PDCD4 expression in gastric cancer tissue and CagA activation of TWIST1 expression and inhibition of E-cadherin and PDCD4 expression in gastric cancer cells.

## Materials and Methods

### Patients and tissue specimens

The study was approved by the Ethics Committee of Shandong University School of Medicine, and all samples and information were gathered with written informed consent. Gastric tumor tissues and their matched non-cancerous tissues were harvested from 30 patients during surgery at Qilu Hospital, Shandong University (Jinan, China) from 2010 to 2012. None of the patients had received adjuvant chemotherapy or radiotherapy before surgery. Diagnosis of gastric cancers was histopathologically confirmed by hematoxylin and eosin staining.

### Cell culture and transfection

Human gastric cancer cell lines (AGS, BGC-823 and SGC-7901) were obtained from the China Academia Sinica Cell Repository (Shanghai). Cells were incubated in the recommended media and a humidified atmosphere containing 5% CO_2_ at 37°C without antibiotics. SGC-7901 cells and BGC-823 cells were cultured in RPMI 1640 medium supplemented with 10% fetal bovine serum (FBS), and AGS cells were cultured in F12 medium supplemented with 10% FBS. Media and FBS were purchased from Invitrogen (USA). Then, cells were transiently transfected with plasmids pCDNA3.1 or pCDNA3.1-CagA (a gift from Dr. Yongliang Zhu, Zhejiang University, China) and pEGFP-C1 or pEGFP-C1-PDCD4 (constructed and provided by Dr. Youhai Chen, Perelman School of Medicine, University of Pennsylvania, Philadelphia, USA) by using X-treme GENE HP Transfection Reagent (Roche Diagnostics, Germany) according to the manufacturer's protocols.

### RNA isolation, reverse transcription and qRT-PCR

Total RNA was extracted from cells by use of Trizol reagent (Invitrogen, USA). Reverse transcription involved the Superscript III First Strand Synthesis kit (Invitrogen). Expression of genes was detected by use of Quantitative real-time PCR SYBR Premix Ex Taq (Takara,China) and normalization involved the 2^−△△ct^ method relative to β-actin. Primer sequences were for PDCD4, sense, 5'-CAGTTGGTGGGCCAGTTTATTG-3' and antisense, 5'-AGAAGCACGGTAGCCTTATCCA-3'; E-cadherin, sense, 5'-AATCCAAAGCCTCAGGTCATAAACA-3' and antisense, 5'-TTGGGTCGTTGTACTGAATGGTC-3'; CagA, sense, 5'-CCGGGGTACCAATTGGAGAGCAGAACCG-3' and antisense, 5'-CCGTCGAGTTAAGATTTTTGGAAACC-3'; and TWIST1, sense, 5'-GGCATCACTATGGACTTTCTCTATT-3' and antisense, 5'-GGCCAGTTTGATCCCAGTATT-3'; vimentin, sense, 5'-CTCTTCCAAACTTTTCCTCCC-3' and antisense, 5'-AGTTTCGTTGATAACCTGTCC-3'; SNAIL, sense, 5'-GGAAGCCTAACTACAGCGAGCT-3' and antisense, 5'-TCCCAGATGAGCATTGGCA-3'; β-actin, sense, 5'-GTGGGGCGCCCCAGGCACCA-3' and antisense, 5'-CTCCTTAATGTCACGCACGATTT-3'.

### Western blot analysis

Protein was extracted from samples and separated by SDS-PAGE, then transferred onto PVDF membranes (Millipore Corp., Billerica, MA, USA), which were blocked immediately with 5% nonfat milk in TBST containing 1% Tween-20 for 1.5 h at room temperature. After being washed in phosphate-buffered saline (PBS) 3 times, membranes were incubated with antibodies against PDCD4 (1∶500; Cell Signaling Technology, USA), TWIST1 (1∶2000, Novus Biologicals, USA), SNAIL (1∶1000, Cell Signaling Technology, USA), CagA (1∶500, Santa Cruz Biotechnology, Santa Cruz, CA), E-cadherin (1∶1000, Cell Signaling Technology, USA), vimentin (1∶1000, Cell Signaling Technology, USA), and β-actin (1∶2000, Sigma-Aldrich, USA), as a normalization control, at 4°C overnight, then with horseradish peroxidase-conjugated secondary antibodies at room temperature for 1 h. After a washing, immunoreactive bands were visualized by enhanced chemiluminescence (Millipore Corp., Billerica, MA, USA). Western blotting was performed 3 times for each sample. Protein was loaded at 20 to 50 µg per lane.

### Immunohistochemistry

Paraffin-embedded tissue specimens were de-paraffinized with xylene and dehydrated with a graded series of alcohol. Antigen retrieval by heat treatment performed in 0.1 M citrate buffer. And blocking the endogenous peroxidase activity was used by 3% H_2_O_2_. Then slides were incubated with antibodies against PDCD4 (1∶200), Twist1 (1∶500), vimentin (1∶100) or E-cadherin (1∶500), and corresponding horseradish peroxidase-conjugated secondary antibodies and DAB for nuclei. Antibodies for immunohistochemical staining were those used for western blot. Finally, hematoxylin was used for counterstaining slides. Tumor differentiation of cancer tissues was determined in a blinded manner. The first antibody was replaced with phosphate buffered saline as negative control. Specimens were viewed under a Nikon Eclipse E800 (Nikon, Tokyo, Japan) microscope.

All stained sections were observed and scored by 2 independent blinded investigators, and slides were given a total staining index (SI) score according to the staining intensity and the percentage of positive tumor cells. Staining intensity was graded as 0, no staining; 1, weak staining; 2,moderate staining; and 3, strong staining. Tumor cell proportion was scored as 0, ≤20% positive tumor cells; 1, 20%–40% positive tumor cells; 2, 40%–60% positive tumor cells; 3, 60%–80% positive tumor cells; and 4, ≥80% positive tumor cells. By assessing the SI, the staining results were finally recorded as 0–4, low expression; 4–6, moderate expression; and 6–12, high expression. If the staining interpretation differed between the investigators, the data for the section were discarded.

### Matrigel invasion assay

Matrigel invasion assay involved a transwell chamber (Costar, New York, USA). SGC-7901 cells were transfected with 2 µg pCDNA3.1, pEGFP-C1; pCDNA3.1-CagA, pEGFP-C1; or pCDNA3.1-CagA, pEGFP-C1-PDCD4. After 48 h, 1×10^5^ post-transfected cells were trypsinized and plated on transwell chambers precoated with 20 µg Matrigel. Medium containing 20% FBS in the lower chamber served as the chemoattractant, and the upper chamber was filled with medium containing 10% FBS. Non-migrated cells were removed with cotton swabs after 24 or 48 h. Cells migrating to the underside of the filter were stained with 0.1% crystal violet and counted on a microscope. Finally, remaining cells were harvested for protein and RNA isolation. Each treatment was repeated 3 times.

### Statistical analysis

Data are expressed as mean ± SD. Student *t* test or chi-square test was used to compare 2 groups. Data analysis involved SPSS 12.0 (SPSS, Chicago, IL, USA). *P* <0.05 was considered statistically significant.

## Results

### 1. Expression of TWIST1, E-cadherin,vimentin and PDCD4 in gastric cancer

**Figure 1:**
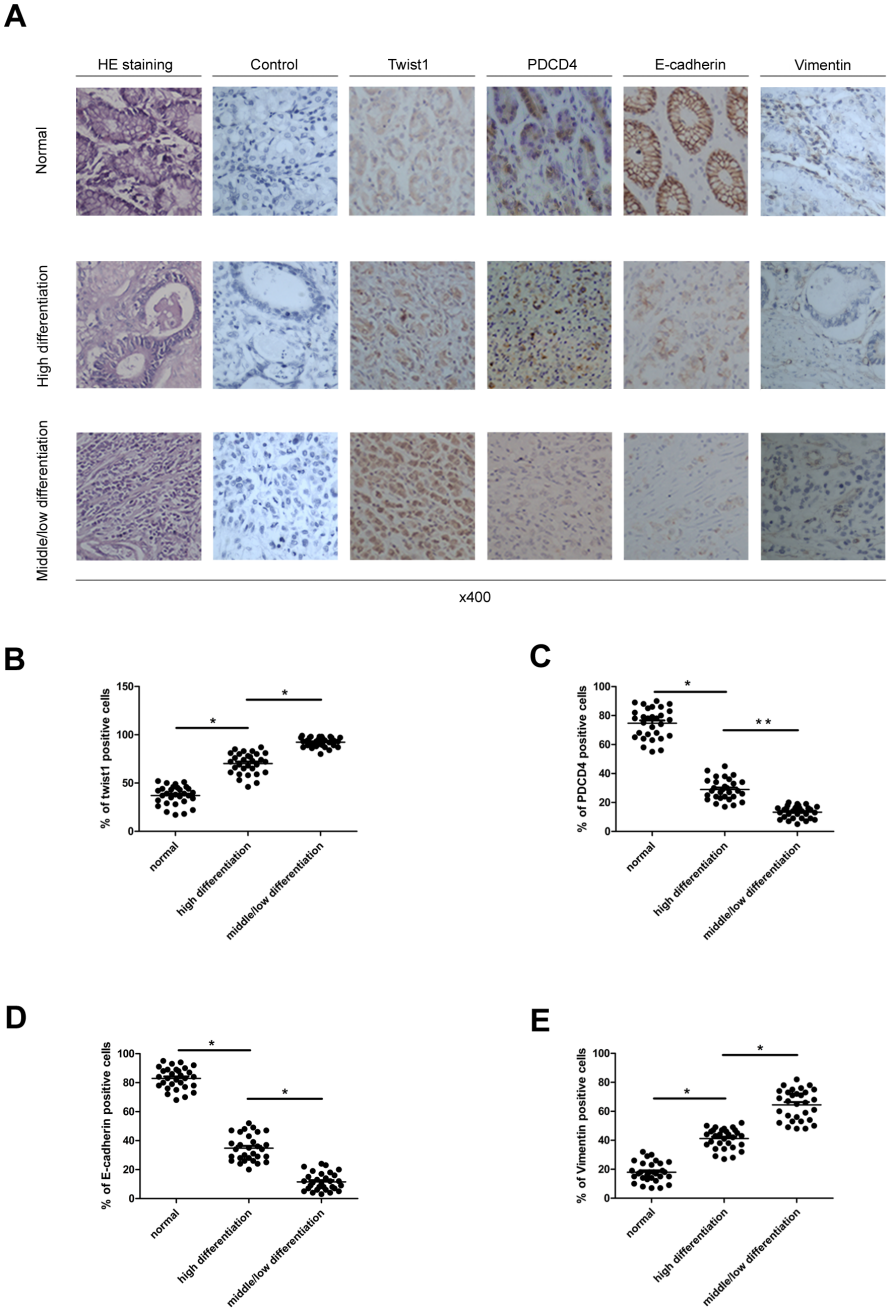
Expression of epithelial–mesenchymal transition (EMT)-related genes and programmed cell death factor 4 (PDCD4) were regulated in human gastric cancer tissues. (A) Hematoxylin and eosin staining confirmed preoperative diagnosis and histological grade. Representative immunohistochemical staining of TWIST1, SNAIL, PDCD4, vimentin and E-cadherin distinct in adjacent normal tissue and high differentiation cancer tissue and middle/low differentiation cancer tissue. (B–E) Quantification of TWIST1-, SNAIL-, PDCD4-, vimentin- and E-cadherin- expression in normal and cancer tissues. The total number of samples is 30 and each point represents 1 sample. Horizontal medium bar is mean and whiskers are SD. ^*^ *P*<0.05 and ^**^ *P*<0.01 versus control.

To investigate the association of EMT and gastric cancer, we harvested 30 clinical tissue samples from gastric cancer patients. Biopsies were divided into middle/low differentiation tissue, high differentiation tissue and non-cancerous tissue adjacent to carcinoma by hematoxylin and eosin staining (**Fig. 1A**). On Immunohistochemistry, TWIST1 and vimentin expression were higher in malignant tissue than non-cancerous tissue, while the expression of E-cadherin and PDCD4 was opposite to that of TWIST1 (**Fig. 1A–E**). TWIST1 and PDCD4 expression was associated with the degree of tumor differentiation but not sex or age (p = 0.021 and p = 0.013, **Table 1**). As well, decreased PDCD4 expression was inversely associated with changed TWIST1 expression during gastric cancer (p = 0.035, **Table 2**). Therefore, EMT may play an important role in gastric carcinogenesis and development.

**Table 1:** Association between TWIST1 or PDCD4 expression and clinicopathological features in gastric cancer patients.

		PDCD4 expression			Twist1 expression		
Characteristic	Total	0–4	6–12	*P-value*	0–4	6–12	*P-value*
Gender							
Men	18	11	7	0.534	6	12	0.472
Women	12	8	4		3	9	
Age							
≥60	23	13	10	0.171	5	18	0.096
<60	7	6	1		4	3	
Tumor differentiation							
Middle/low	14	12	2	0.021	1	13	0.013
High	16	7	9		8	8	

Pathology rating criteria: **1)** immunohistochemistry positive expression rate: 0 points <20%, 1 points 20–40%, 2 points 40–60%, 3 points 60–80%, 4 points >80%; **2)** protein color intensity: 0, no; 1 point, weak; 2 points, medium; 3 points, high. Rate for the protein expression of tissues with 2 sections. Scores were 0, 1, 2, 3, 4, 6, 8, 9 and 12. 0–4, low expression; 6–12, high expression.

**Table 2:** Association between expression of Twist1 or PDCD4 in gastric cancer.

			Twist1 expression		
Variable		Total	0–4	6–12	*P-value*
PDCD4 expression					
0–4		19	3(15.8%)	16(84.2%)	0.035
6–12		11	6(54.6%)	5(45.4%)	

Pathology rating criteria: **1)** immunohistochemistry positive expression rate: 0 points <20%, 1 points 20–40%, 2 points 40–60%, 3 points 60–80%, 4 points >80%; **2)** protein color intensity: 0, no; 1 point, weak; 2 points, medium; 3 points, high. Rate for the protein expression of tissues with 2 sections. Scores were 0, 1, 2, 3, 4, 6, 8, 9 and 12. 0–4, low expression; 6–12, high expression.

### 2. TWIST1, vimentin or E-cadherin expression in gastric cell lines is regulated by CagA

**Figure 2:**
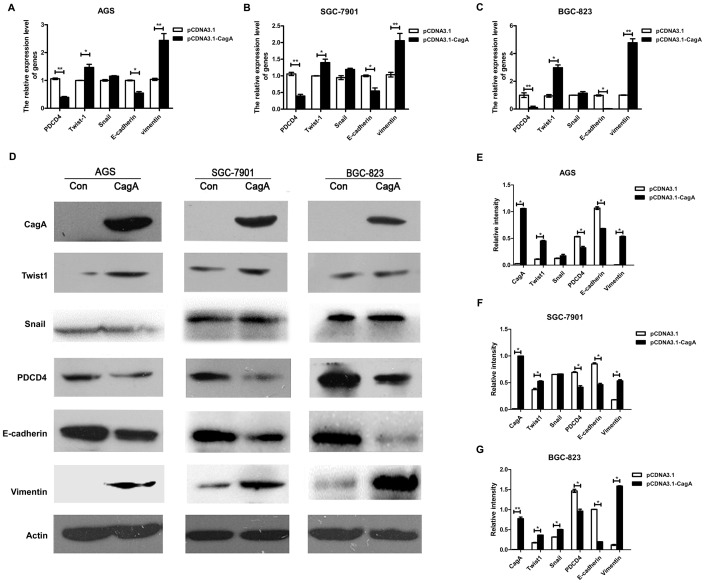
Expression of TWIST1, SNAIL, PDCD4, vimentin and E-cadherin were regulated by *H. pylori* cytotoxin-associated gene A (CagA) in gastric cancer cells. CagA full-length plasmid was transfected into AGS, SGC-7901 and BGC-823 cells for 48 h,and cells were harvested for mRNA and western blot analysis. (A–C) Quantitative RT-PCR analysis of mRNA level of PDCD4, EMT-related transcription factors (TWIST1 and SNAIL) and cell markers(vimentin and E-cadherin)in 3 gastric cancer cell lines. All values were normalized to β- actin mRNA in the same sample. (D) Western blot analysis of protein level of CagA, TWIST1, SNAIL, PDCD4, vimentin and E-cadherin in control or CagA-transfected cells. (E–G) Western blot densitometry was quantified (ImageJ) and the level of the indicated protein was normalized to β-actin. Data shown represent mean±SD of 3 independent experiments. * *P*<0.05,** *P*<0.01 versus control.

Next, we explored whether CagA, as a virulent factor of *H. pylori*, influenced the EMT and therefore promoted the invasion and metastasis in gastric cancer. We chose 3 human gastric cancer cell lines of differentiation statuses (AGS, well-differentiated gastric epithelial cell line; SGC-7901, moderately differentiated cell line and BGC-823 poorly differentiated cell line). CagA expression plasmid was transfected into various gastric cell lines. Although the morphological changes of CagA-transfected cells were not observed (data not shown), the expression of transcription factors and cell markers were affected by CagA. The mRNA and protein expression of TWIST1 and vimentin was upregulated with CagA transfection (**Fig. 2A–G**) and that of PDCD4 and E-cadherin was downregulated (**Fig. 2A–G**), but no significant changes in SNAIL expression were observed in CagA transfection group. Therefore, *H. pylori* CagA may affect EMT regulators including TWIST1. Moreover E-cadherin, as an important epithelial marker and vimentin, as a mesenchymal marker regulated by TWIST1, were also influenced by CagA, and the EMT-related gene PDCD4 was downregulated.

### 3. Expression of TWIST1, vimentin and E-cadherin was influenced by PDCD4

**Figure 3:**
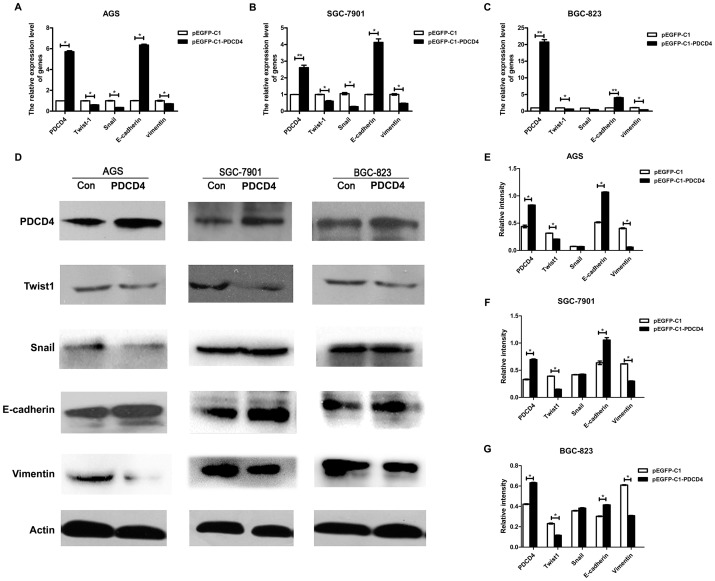
PDCD4 regulated EMT-related transcription factors and cell markers in gastric cancer cell lines. Plasmids of pEGFP-C1 and pEGFP-C1-PDCD4 were respectively transfected into AGS, SGC-7901 and BGC-823 cells for 48 h, and then cells were harvested for isolating total cellular RNA or protein. (**A–C**) qRT-PCR analysis of mRNA level of TWIST1, SNAIL, PDCD4, vimentin and E-cadherin in AGS, SGC-7901 and BGC-823 cells. All values were normalized to β-actin mRNA in the same sample. (D) Western blot analysis of 5 target proteins expression in control cells and PDCD4 overexpression cells. (E–G) Western blot results were quantified (ImageJ) and the level of the target protein was normalized to β-actin. Data are mean±SD from 3 independent experiments. * *P*<0.05,** *P*<0.01 versus pEGFP-C1 con.

We next analyzed the molecular mechanism of the EMT regulated by *H. pylori* CagA in gastric cancer cells. PDCD4 suppresses cell growth via a direct interaction with TWIST1 in human prostate cancer [22]. Immunohistochemistry and statistical analysis demonstrated the relevance of PDCD4 and TWIST1 existed in gastric carcinoma (Fig. 1, Table 2). However, the mechanisms underlying PDCD4 function in gastric cancer cells were not clear. So AGS?GSC-7901 and BGC-823 cells we transfected with a plasmid of overexpressing PDCD4. PDCD4 mRNA and protein expression was upregulated in all plasmid-transfected cell lines (**Fig. 3A–G**). In addition, mRNA and protein levels of TWIST1 and vimentin, but not SNAIL, were decreased to various degrees, and E-cadherin levels were increased (**Fig. 3A–G**). Therefore, TWIST1, vimentin and E-cadherin expression may be regulated by PDCD4 in gastric epithelial cells.

### 4. The EMT is regulated by CagA via inhibiting PDCD4 in gastric epithelial cells

**Figure 4:**
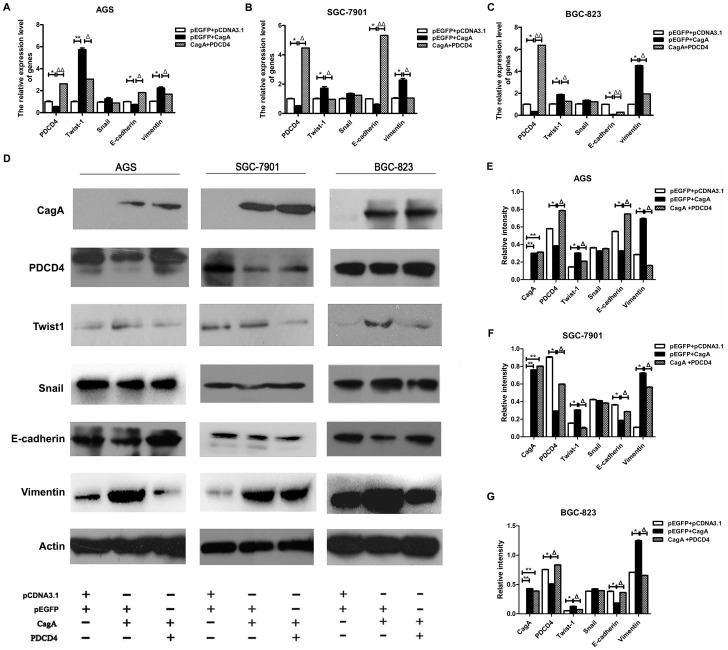
*H. pylori* CagA promoted EMT in part by regulating PDCD4. All experiments were divided into three groups: 1) pCDNA3.1+pEGFP-C1 (control), 2) pCDNA3.1-CagA+pEGFP-C1, 3) pCDNA3.1-CagA+pEGFP-C1-PDCD4. According to various groups, the corresponding plasmids were transfected into AGS, SGC-7901 and BGC-823 cells. Then cells were harvested for mRNA and western blot analysis. (A–C) qRT-PCR analysis of mRNA level of TWIST1, SNAIL, PDCD4, vimentin and E-cadherin in gastric cancer cell lines. All values were normalized to β- actin mRNA in the same sample. (D) Western blot analysis of CagA, TWIST1, SNAIL, PDCD4, vimentin and E-cadherin protein expression in all cells. (E–G) Western blot results were quantified (ImageJ) and normalized to β-actin. Data shown represent mean±SD from 3 independent experiments. * *P*<0.05, ** *P*<0.01 versus con, ^Δ^ *P*<0.05,^ΔΔ^ *P*<0.01 versus pEGFP + CagA.

**Figure 5:**
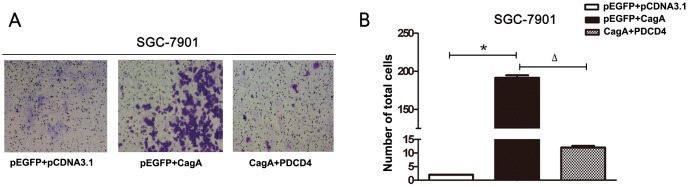
Cell migration assays of PDCD4 overexpression downregulating CagA-induced EMT biological phenomena. (**A**) The experiment was divided into three groups: 1) pCDNA3.1+pEGFP-C1 (control), 2) pCDNA3.1-CagA+pEGFP-C1, 3) pCDNA3.1-CagA + pEGFP-C1-PDCD4.The corresponding plasmids were transfected into SGC-7901 cells according to various groups. The invasion and metastasis ability of cells was analyzed by matrigel invasion assay. (**B**) Quantitative the migrated cell numbers of SGC-7901 in three groups. Data shown represent mean±SD from 3 experiments. * *P*<0.05 versus con, ^Δ^ *P*<0.05 versus pEGFP + CagA.

Based on the above findings, we hypotheses that the EMT process induced by *H. pylori* CagA occurred by downregulating PDCD4, we cotransfected gastric epithelial cells with CagA plasmid and PDCD4 overexpression plasmid. The decreased expression of PDCD4 was recovered with CagA and overexpressed PDCD4 transfection. As well, the increased expression of TWIST1 and vimentin was inhibited and the decreased expression of E-cadherin was upregulated in the co-transfected group. These changes occurred at both the mRNA and protein levels (**Fig. 4A–G**). The migration of human gastric cancer cell line SGC-7901 transfected with plasmids was determined by matrigel invasion assay. Results show that it was induced by CagA plasmid. Cell migration assay also revealed that PDCD4 overexpression downregulated CagA-induced their invasion and metastasis ability (**Fig. 5A**). Fig. 5B shows the migrated cell numbers of SGC-7901 in CagA transfection group are obviously more than control group, while the amount of migrated cell in co-transfection group is significantly less than CagA transfection group. The biological phenomena demonstrate EMT promotion by CagA was recovered in part by elevated PDCD4 expression. On the basis of evidence, it inferred that PDCD4 may be required for *H. pylori* CagA-mediated gastric cancer progression.

## Discussion

The main findings of this study are summarized as follows: 1) *H. pylori* CagA is responsible for induction of TWIST1 or vimentin and inhibition of E-cadherin in gastric cancer cells. 2) CagA-induced epithelial-mesenchymal transition is in part dependent on regulating PDCD4. 3) TWIST1 and PDCD4 involves in the EMT of gastric cancer.

Gastric cancer is a multi-step and gradual disease. Excess proliferation of epithelial cells is an important part of the initial tumor angiogenesis or early growth [5]. Subsequently, cell invasion, initially reflected through the basement membrane, was considered a predictor of the final stage of cancer; it eventually leads to metastatic spread and mortality [26]. Pathogenic infection could cause cancer tumorgenesis and malignant transformation of host cells. As an important pathogenic risk factor, *Helicobacter pylori* were closely related to peptic ulcer disease, mucosa-associated lymphoid tissue lymphoma of stomach, and gastric adenocarcinoma [20], [27], [28], [29]. CagA, one of the most principle virulence factors of *H. pylori*, induces occurrence and development of gastric cancer. Malignant transformation of host cells involves in regulationary network, including numerous cell proteins, non- coding RNA, and other biological activity molecules. However, the mechanism underlying CagA effects on the gastric mucosa is so complex and unclear.

The EMT is a highly conserved biological process on embryonic organ development, which allows epithelial cells to lose epithelial phenotype and obtain mesenchymal phenotype. This includes loss of cell polarity in epithelial cells, loss of the ability to connecting with basement membrane, access to mesenchymal phenotype such as higher migration and invasion, anti-apoptosis, and the ability to degrade the extracellular matrix [8], [30]. EMT plays an essential role in chronic inflammatory diseases, fibrotic diseases, tumor progression and tissue reconstruction process, especially in the acquisition of migration and invasion ability for malignant epithelial cells. Abnormal activity in the EMT epith of adult epithelial cells inhibited cell adhesion molecules, caused decreased cell adhesion capacity, and thereby enabled tumor cells to spread in the body, ultimately promoting tumor metastasis [31], [32], [33]. Therefore EMT is considered the beginning of the invasion and metastasis and is also a sign of strong ability of invasion and metastasis of tumor cells. *H.pylori* induces epithelial-mesenchymal transition by TNF-α inducing protein[34]. The current study indicates CagA can lead epithelial cells to morphological changes. It has been proposed that CagA expression was not only sufficient to disrupt apical junctions but also resemble epithelial to mesenchymal transition in Madin-Darby canine kidney cells [35]. Epithelial cells infected by the bacteria appeared various genomics changes, and the effect on EMT markers induced by *H. pylori* was probably on a cagPaI-related manner. Data showed that epithelial mesenchymal transition (EMT)-related genes were up- or down-regulated by CagA-positive *H. pylori* strains in AGS [36], [37]. Of note, the regulatory network of CagA involving in the EMT of gastric cancer is still need to be clarified.

To investigate the specific mechanism of EMT promoted by CagA in gastric cancer, we compared genes expression of adjacent non-tumor tissues and gastric carcinoma tissues by immunohistochemistry. Results indicated that Twist1/vimentin expression in carcinoma tissues were higher than adjacent non-tumor tissues, and its expression in middle/low differentiation carcinoma tissues higher than high differentiation tissues. However, E-cadherin and PDCD4 decreased in the process of epithelial-mesenchymal transition in gastric cancer. Interestingly, it exists on a negative correlation to the expression of TWIST1 and PDCD4. Our study suggested EMT-related genes are associated with the process of gastric cancer, consistent with previous researches observing loss of epithelial protein and/or acquisition of the expression of mesenchymal proteins occurred in gastric carcinoma. Furthermore, they were respectively correlated to poorly differentiated histology, advanced stage and poor patient outcome [38]. In addition, PDCD4 is a newly found molecule that plays a vital role on many biological processes that may cause disease or occurrence and development of tumor. PDCD4 inhibits the translation process by binding to translation initiation factor eIF4A or eIF4G. Different inducers of apoptosis stimulate cells and upregulate the expression of PDCD4, which could further regulate P21, Cdk4, carbonic anhydrase II and JNK/c-Jun/AP-1. PDCD4 could inhibit invasion-related urokinase plasminogen activator receptor expression, invasion or infiltration of blood vessels, and metastasis; therefore, decreased expression of PDCD4 plays an important role in tumor occurrence, development and prognosis [17], [33], [39], [40]. So, we wondered if Twist1 and PDCD4 participated in the CagA-induced EMT.

Then, we detected EMT-associated transcription factors and markers expression in CagA-transfected gastric cancer cell lines. Our data showed that *H. pylori* CagA could up-regulate expression of TWIST1 and vimentin but not SNAIL, and down regulate E-cadherin and PDCD4 in gastric cell lines (AGS, SGC-7901 and BGC-823). The results were not affected by the differentiation status of transfected cell. Although we did not observe the morphological changes of CagA-transfected cells, invasion and metastasis ability of gastric cancer cells could be accelerated by CagA via transwell cell invasion assay. This is in part caused by the functional heterogeneity of CagA in different tissues. So the effect of EMT induced by *H. pylori* still needed to be further explored. MiR-21 promoted TGF-β–induced myofibroblast differentiation process by targeting PDCD4. TGF-β induced miR-21 expression in fibroblasts and mir-183 downregulated PDCD4 and inhibited TGF-β–induced apoptosis in human hepatocellular carcinoma cells [41], [42]. Meanwhile, TGF-β regulated SNAIL, TWIST, or ZEB1 and affected the EMT and metastasis [43]. Moreover, PDCD4 was found to be involved in transcription too. Indeed, the COOH terminus of Twist1 containing the basic helix-loop-helix domain was suggested that mediated the direct interaction with PDCD4 proteins. So, PDCD4 interacts with the DNA binding domain of Twist1 and inhibits its DNA binding ability to target genes in human prostate cancer cells [22].Therefore, we further tested whether PDCD4 could regulate TWIST1 in gastric cancer cells. Our study showed that TWIST1 was down regulated by PDCD4 high expression plasmid by real-time PCR and western blotting, while E-cadherin expression was opposite to TWIST1 in AGS, SGC-7901 and BGC-823. The mRNA expression of SNAIL was downregulated in AGS and SGC-7901, while preotein expression was not observed the same changes. It may be due to the complexity of protein synthesis. That is to say, protein is not only regulated on transcription level but also translation and turn over level. It occasionally appears that mRNA is not a direct indication of protein level in cells. Last, we carried out further research on the role of PDCD4 in CagA-induced epithelial-mesenchymal transition. Gastric epithelial cells were transfected with CagA expression plasmid and PDCD4 overexpression plasmid simultaneously. Restoration of PDCD4 expression could inhibit TWIST1 and vimentin, and induce E-cadherin which regulated by CagA, PDCD4 was also certified that could affect the invasion and metastasis ability in gastric cells by matrigel invasion assay. Although additional studies are necessary to test this hypothesis, we concluded basically that CagA-induced epithelial-mesenchymal transition is in part dependent on regulating PDCD4.

**Figure 6:**
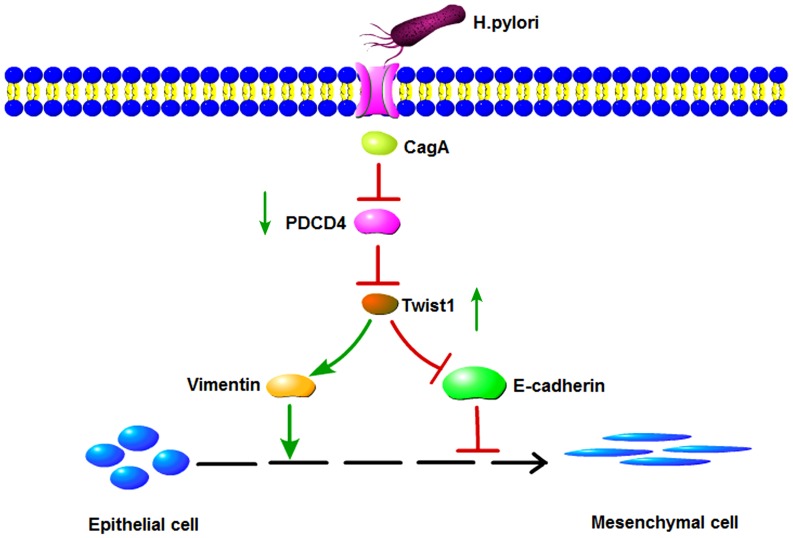
Schematic representation of the signaling pathways involved in the *H. pylori* CagA -induced epithelial-mesenchymal transition in gastric cancer cells. *H. pylori* virulence factors CagA suppressed PDCD4 expression in three gastric cancer cell lines, while the decline of PDCD4 could accelerate transcription factor TWIST1 expression. And then the latter regulates epithelial cell marker (E-cadherin) and mesenchymal cell marker (vimentin). Ultimately,epithelial-mesenchymal transition in gastric cancer cells was induced in the process.

In summary, we demonstrate that *H. pylori* CagA induce TWIST1 expression and EMT in gastric cancer cells by regulating PDCD4 (Fig. 6). We provide some insight into the molecular network of gastric cancer induced by *H. pylori* infection, and supply a theoretical basis for PDCD4 as a biological target for diagnosis or treatment of cancer. Future research involving mouse models may lead to a better understanding of the role of PDCD4 in the *H. pylori*-induced EMT of gastric cancer.
